# Association of Neutrophil–Lymphocyte and Platelet–Lymphocyte Ratio with Adverse Events in Endovascular Repair for Abdominal Aortic Aneurysm

**DOI:** 10.3390/jcm10051083

**Published:** 2021-03-05

**Authors:** Maria P. Ntalouka, Petroula Nana, George N. Kouvelos, Konstantinos Stamoulis, Konstantinos Spanos, Athanasios Giannoukas, Miltiadis Matsagkas, Eleni Arnaoutoglou

**Affiliations:** 1 Department of Anesthesiology, Faculty of Medicine, School of Health Sciences, University of Thessaly, 41110 Larissa, Greece; konstaarist@gmail.com (K.S.); earnaout@gmail.com (E.A.); 2 Department of Vascular Surgery, Faculty of Medicine, School of Health Sciences, University of Thessaly, 41110 Larissa, Greece; petr.nana7@hotmail.com (P.N.); geokouv@gmail.com (G.N.K.); spanos.kon@gmail.com (K.S.); agiannoukas@hotmail.com (A.G.); milmats@gmail.com (M.M.)

**Keywords:** aortic aneurysm, abdominal, endovascular procedures, biomarkers/blood, blood platelets/surgery, lymphocyte/surgery, acute kidney injury

## Abstract

The association of chronic inflammatory markers with the clinical outcome after endovascular aneurysm repair (EVAR) for abdominal aortic aneurysm (AAA) was investigated. We included 230 patients, treated electively with EVAR. The values of neutrophil–lymphocyte ratio (NLR) and platelet–lymphocyte ratio (PLR) were measured pre- and postoperatively. Any major adverse cardiovascular event (MACE) and acute kidney injury (AKI) were recorded. Adverse events occurred in 12 patients (5.2%). Seven patients suffered from MACE and five from AKI. Median NLR and PLR values were significantly increased after the procedure (NLR: from 3.34 to 8.64, *p* < 0.001 and PLR: from 11.37 to 17.21, *p* < 0.001). None of the patients or procedure characteristics were associated with the occurrence of either a MACE or AKI. Receiver operating characteristic curve analysis showed that postoperative NLR and PLR were strongly associated with AKI. A threshold postoperative NLR value of 9.9 was associated with the occurrence of AKI, with a sensitivity of 80% and specificity of 81%. A threshold postoperative PLR value of 22.8 was associated with the occurrence of AKI, with a sensitivity of 80% and specificity of 83%. Postoperative NLR and PLR have been associated with the occurrence of AKI after EVAR for AAA.

## 1. Introduction

Abdominal aortic aneurysm (AAA) repair is a well-recognized high-risk surgical procedure. AAA is characterized by a systemic inflammatory response (SIRS) mainly due to extensive hormonal and metabolic stress response activation [1,2,3,4,5,6,7]. In general, the risk of SIRS is considered to be lower with the implementation of endovascular aneurysm repair (EVAR) in contrast to open repair (OR), mainly due to the less intensive and extensive inflammatory cascade and cytokine production and less tissue damage and ischemia-reperfusion insult [2,7].

However, in patients with AAA treated by EVAR, the manipulations within the aortic lumen and presence of intramural thrombus may exacerbate SIRS [2,7]. The endovascular technique itself may influence the interaction between the graft material and endothelium and may aggravate endothelial dysfunction and thus intensify the inflammatory reaction [1,2,3,4,5,6,7]. Early research suggested that in some patients, harmful levels of cytokines, especially IL-6, were reached after EVAR [7]. The systemic inflammatory response associated with EVAR is known as postimplantation syndrome (PIS) and may negatively affect the 30-days postoperative outcomes [8].

The neutrophil to lymphocyte ratio (NLR) and platelet to lymphocyte ratio (PLR) have been increasingly recognized as biomarkers of systemic inflammation [9]. Moreover, they could predict the outcome in patients suffering from a variety of conditions, including major abdominal and cardiac surgery and percutaneous coronary intervention [9,10,11,12,13,14,15,16,17,18,19,20,21,22]. Recently, the role of NLR and PLR has been investigated in vascular surgery with limited data existing regarding their role in the early postoperative period [23,24,25,26,27,28,29,30]. The present study was designed to investigate the association of inflammatory markers NLR and PLR with the clinical outcome after EVAR for AAA during the early follow-up.

## 2. Materials and Methods

### 2.1. Study Cohort

A retrospective analysis of prospective data of consecutive patients treated electively with standard EVAR for infra-renal AAA, using currently available endografts (Medtronic Endurant, Santa Ana, CA, USA; Cordis Incraft, Dublin, OH, USA; Treovance Bolton, Sunrise, FL, USA; Gore Excluder, W.L. Gore and associates, Flagstaff, AZ, USA; Endologix AFX 2, Irvine, CA, USA, COOK, Zenith, Bloomington, IN, USA; Jotec, E-tegra, Hechingen, Germany) was undertaken in a single tertiary center from March 2016 to February 2019. Patients were treated mainly based on the European Society of Vascular Surgery (ESVS) guidelines [31,32]. However, the final decision on patients’ management was in the surgeon’s discretion and thus, in some cases, the endograft selection was excluded from instructions for use. Exclusion criteria included:

- Clinical and/or laboratory evidence of infection preoperatively, including leukocytosis (white blood cell count (WBC) > 10.000/mL) and elevated body temperature.
- Signs of gangrene.
- Previous trauma or surgery two months prior to enrolment.
- Any autoimmune disease or systemic inflammatory condition.
- Any malignancy.

A dedicated database existed for the prospective collection of patients’ data, including demographics (age, sex), comorbidities, hypertension, dyslipidemia, tobacco use at any time, chronic renal failure (according to KDIGO criteria [33], glomerular filtration rate (GFR) < 60 mL/h/1.73 m^2^, estimated with the Cockcroft–Gault equation [34]), hemodialysis, coronary artery disease (previous myocardial infarction, percutaneous transcatheter coronary angioplasty, coronary–aortic bypass), diabetes mellitus, chronic obstructive pulmonary disease and intraoperative details (blood transfusion, type of anesthesia, intravenous contrast use, intraoperative technical complications, renal artery occlusion). Laboratory exams were preoperatively (within 24 h before the operation) and postoperatively (within 24 h after surgery) recorded. Blood tests included the values of hemoglobin, white blood cells (neutrophils, lymphocytes and NLR), platelets (PLR), urea, creatinine and CRP.

Early postoperative follow-up included computed tomography angiography (CTA) at the first month and clinical and laboratory evaluation. Adverse events such as major adverse cardiovascular events (MACE), acute kidney injury (AKI), limb thrombosis, postoperative infections (trauma, respiratory or urinary tract), PIS, and deaths of any cause were recorded. This study involved the collection of existing data and diagnostic tests that have been recorded in such a manner that subjects could not be identified, either directly or through identifiers linked to the subject. The study was approved by the Scientific Board of University General Hospital of Larissa, Greece (42627, 4 October 2019) and was registered (NCT04254211).

### 2.2. Definitions

Under the term MACE, myocardial infarction, arrhythmia and stroke, including transient ischemic attack, were included. Myocardial infarction was recorded at any new electrocardiographic sign or biochemical marker elevation (high sensitivity troponin) signaling myocardial ischemia. Arrhythmia was considered any event of atrial or ventricular tachycardia (more than 90 pulses per minute) or any episode of bradycardia of less than 50 pulses per minute. AKI was defined according to the RIFLE (Risk, Injury, Failure, Loss of kidney function, and End-stage kidney disease) criteria [35], as a two-fold increase in serum creatine (Scr) or a decrease in GFR (estimated with the Cockcroft–Gault equation [34]) of more than 50%. Follow-up included the early postoperative 30-day period. The preoperative NLR and PLR values were representing the values within 24 h preoperatively while the postoperative values were within 24 h after the repair.

### 2.3. Outcomes

The primary outcome was the association of the preoperative values of NLR and PLR with the postoperative adverse events (MACE and AKI) in EVAR patients during follow-up. The association of the postoperative values of NLR and PLR with adverse events during follow-up was defined as the secondary outcome.

### 2.4. Statistical Analysis

Data are expressed as mean ± standard deviation except for non-Gaussian parameters that are presented as median and interquartile range. Categorical data were expressed as absolute numbers and percentage of prevalence (%) in the study cohort. In the statistical analysis for continuous variables, the independent t-test for normally distributed data and the Mann–Whitney U test for nonparametric data were used. Univariate and multivariate Cox proportional hazard regression analysis was used to evaluate the effect of relevant patients’ or procedural risk factors for cardiovascular events or AKI occurrence. *p* value was considered significant when it was <0.05. Statistical analysis was performed by SPSS 22.0 for Windows software (IBM Corp, Armonk, NY, USA).

## 3. Results

In total, 242 consecutive patients treated electively with EVAR were included in the analysis. Twelve patients with abnormal preoperative WBC count were excluded from the study. Eight patients had cancer, two patients suffered from rheumatoid arthritis, while in the remaining two, there were no clinical signs of inflammation or malignancy. Finally, 230 patients were included in the study. The median age was estimated at 72.1 years (range 64–82). Males were 228 (99.1%). The mean AAA diameter was estimated at 58.6 ± 9 mm (range 51.9–62.8 mm). In total, 18 patients suffered from renal insufficiency (GFR < 60 mL/min/1.73 m^2^) while one patient was under hemodialysis preoperatively. Hypertension was the most common comorbidity (83.9%), followed by dyslipidemia (79.6%). All preoperative patients’ characteristics are presented in detail in Table 1. Regarding graft application, 77 patients were treated using the Endurant endograft, 49 with Excluder, 53 with AFX 2, 21 with Treovance, 15 with Incraft, 9 with E-Tegra and 6 with Zenith. In all cases, except the patients treated with endovascular sealing device (Endologix Nellix, Irvine, CA, USA), a standard bifurcated endograft was used.

The majority of patients were operated under general anesthesia (83.4%). The need of transfusion per patient was estimated at 0.5 units of red blood cells while 55 patients needed a transfusion intraoperatively. One accessory renal artery with a diameter of less than 3 mm was intentionally covered without any impact on renal function. In two cases, a cranial migration of the main body of the endograft during the procedure led to renal artery occlusion. Both patients were managed using renal artery stenting. Completion angiography confirmed renal artery patency. No impact on their renal function was recorded. The mean duration of operation was 144 min (range 70–192 min) while the mean intraoperative contrast use per patient was 107 mL (range 65–140).

All patients underwent pre- and postoperative laboratory evaluation at the day before surgery and at day 1 postoperatively. The median hemoglobin value was 13.7 preoperatively and decreased to 11.7 mg/dL postoperatively. Similarly, the preoperative median platelet value was 220.000/μL and decreased to 169.000/μL postoperatively. The available laboratory findings are presented in detail in Table 2.

Total cardiovascular and renal adverse events occurred in 12 patients (5.2%) during the early follow-up. Seven patients suffered from a cardiovascular adverse event and five patients from acute kidney injury. Regarding cardiovascular events, three patients suffered a myocardial infarction, three a new episode of new arrhythmia and one patient suffered a minor stroke. All patients with arrhythmia presented atrial fibrillation. No patient presented a more severe renal defect and/or needed hemodialysis. All patients that presented AKI at the early follow-up were discharged with a normal creatinine value. No clinical signs of renal injury were detected during the postoperative period. All postoperative adverse events are presented in Table 3. None of the patients’ anatomical or procedure characteristics, including type of anesthesia and endograft, were associated with the occurrence of either a MACE or AKI. Median NLR and PLR values were significantly increased after the procedure (NLR: from 3.34 to 8.64, *p* < 0.001 and PLR: from 11.37 to 17.21, *p* < 0.001).

**Figure 1:**
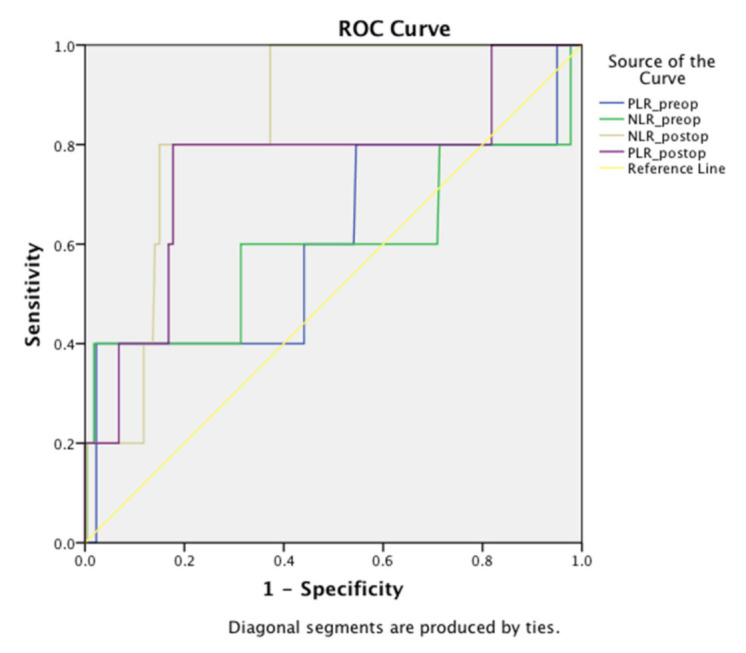
Receiver operating characteristic curve for NLR and PLR with respect to AKI. NLR: neutrophil–lymphocyte ratio; PLR: platelet–lymphocyte ratio; Preop: preoperative; Postop: postoperative; AKI: acute kidney injury; ROC: receiver operating characteristics.

Areas under the curve for preoperative values of NLR and PLR were 0.595 (*p* = 0.46) and 0.604 (*p* = 0.426). None of the preoperative NLR and PLR values were predictive for the occurrence of MACE or AKI (Supplementary Tables S1 and S2). Receiver operating characteristic curve analysis showed that postoperative NLR and PLR were strongly associated with acute kidney injury after EVAR (area under the curve, NLR: 0.843; *p* = 0.009 and PLR: 0.754, *p* = 0.05). A threshold postoperative NLR value of 9.9 was highly associated with the occurrence of AKI, with a sensitivity of 80% and specificity of 81%. A threshold postoperative PLR value of 22.8 was highly associated with the occurrence of AKI, with a sensitivity of 80% and specificity of 83% (Figure 1).

## 4. Discussion

The current study shows that postoperative NLR and PLR are associated with AKI after the EVAR procedure. However, neither NLR nor PLR were associated with the occurrence of any MACE. In a study by Bath et al., elevated postoperative NLR was independently associated with worst postoperative outcome, including renal failure, in patients’ AAA repair [28]. Although in the study by Bath et al. only 20% of the patients were treated with OR, and even though EVAR is associated with less inflammation when compared to OR, the increased inflammatory response may have acted as a confounding factor [2,7,28]. In our study, all patients were treated with EVAR, minimizing the risk of confounding factors regarding the more extensive inflammatory and stress related response [5,7].

An increased preoperative NLR has been associated with increased mortality after EVAR [36]. Moreover, authors suggested that an elevated preoperative NLR value, with a cut-off ≥4.0 could be used to recognize patients with increased risk of postoperative mortality, irrespective of other comorbidities [36]. In our study, there was no association between the preoperative NLR or PLR values and the postoperative morbidity or mortality. However, one preoperative value of NLR does not stand for the individual’s overall stage of health. Moreover, a change in patient’s condition, that may have affected the outcome, may have occurred in the time interval between the blood regimen and the operation.

In the study by Gameiro et al., postoperative NLR and PLR ratios were independently associated with AKI after major abdominal surgery [37]. However, no association was proven between the aforementioned ratios with the in-hospital mortality, as in our cohort, and a possible explanation could be the small sample size [37]. In 2018, Parlar and Saskin showed that both pre- and postoperative NLR and PLR are independent biomarkers for AKI in the early postoperative period following coronary artery bypass grafting (CABG) [12]. Based on our study, a postoperative threshold NLR of 9.9 (80% sensitivity, 81% specificity) and a postoperative threshold PLR of 22.8 (80% sensitivity, 83% specificity) are associated with the occurrence of AKI in the early postoperative period after elective EVAR.

AKI is a costly postoperative complication after EVAR that may negatively impact patient’s prognosis [38,39]. EVAR procedures expose the patient to various risk factors for AKI occurrence, such as microembolization [38,39,40,41]. Although AKI after EVAR has been considered a self-limited condition, it has now been associated with short- and long-term consequences, mainly in terms of mortality [38,39,40]. Moreover, even in high-income countries, postoperative AKI is strongly related with the development of chronic kidney disease (CKD), end-stage kidney disease (ESKD) and death. Of note, despite the advances in our understanding about long-term risks following AKI in high income countries, when compared to low- and middle-income countries (LMICs), substantial gaps in knowledge still remain about effective interventions that could improve the outcome of patients [39].

Today, AKI has been established as a rapidly evolving growing problem with significantly increased risk of unfavorable long-term prognosis. In addition, experts suggest that a singular focus on serum creatinine as a reference standard for acute kidney injury has brought to surface several critical interpretative challenges. The most apparent drawbacks that ensue from the use of serum creatinine as a singular reference standard for early AKI diagnosis are focused mainly on sensitivity and specificity and have been widely acknowledged [39,40]. Based on current research, serum creatinine may variably delay increasing after the kidney injury depending predominantly on renal reserve, supporting the hypothesis that substantial tubular damage could occur before serum creatinine arises. Lastly, during the last few years, several renal-specific factors and a number of patient-related independent contributors, including prerenal azotemia, rhabdomyolysis, medications and decreased creatinine production have been recognized as being responsible for the delayed and the variable alterations in serum creatinine [40].

Of note, recent animal studies that aim for the identification of innovative AKI biomarkers, indicate that leukocytes mobilize from spleen in response to AKI and that neutrophils, monocytes and B cells form an early immune infiltrate into the kidney in the first few hours following AKI [41]. Moreover, an expansion of leukocytes, including T cells, in the kidney during AKI because of both migration and local proliferation has been described [41,42]. Nonetheless, renal B cells produce the chemokine CCL7 which promotes neutrophil and monocyte recruitment, thus exacerbating AKI severity [41]. All the above support the role of inflammation in the development of AKI.

Based on the aforementioned laboratory findings, the CCL7 chemokine was further studied in order to identify its clinical relevance in terms of AKI diagnosis and treatment or prevention. In three independent cohorts of human patients with AKI, significantly higher transcripts and urine levels of CCL7 were observed compared with controls, respectively [41]. Hence, experts highlight the clinical importance of the contribution of the B cells in the early sterile inflammation in AKI via the production of leukocyte-recruiting chemokines [41]. More specifically, it seems that urinary CCL7 may present a useful biomarker for AKI, while the specific blockade of the CCL7 chemokine in the blood could prove to be a useful strategy to reduce the inflammatory kidney infiltration, thereby ameliorating AKI without affecting the rest of the inflammatory cells that fight infections [41,42].

However, it should be noted that CCL7 urine biomarkers and the CCL7 serum blockers are still quite expensive and not available in every hospital. Thus, as the limitations and the delayed response of the serum creatinine are being further acknowledged and the role of inflammation in the development of AKI is more and more recognized, the need for the development of new, cheap and easily available markers that may promptly give rise to suspicions of AKI proves to be of the utmost importance. Moreover, several clinical data suggesting the possibility that a significant amount of candidate markers may be more sensitive than creatinine to kidney injury have also emerged [41,42]. Based on the results of our study, it seems that NLR and PLR could serve as the putative markers that could identify the subgroup of patients that need extensive monitoring, advanced care and experts’ approach for AKI prevention and/or subsequent treatment. This could be in accordance with the current literature that emphasizes the clinical value of new, more reliable, cost-effective, clinically available and easily and quickly obtained markers in order to raise high suspicion and monitor the patients in terms of early recognition of those who are at risk for AKI development [11,12,23,40]

Our study is the first to examine the association between NLR, PLR and postoperative outcome after EVAR, highlighting the importance of perioperative stress response and inflammation. However, these results should be interpreted in the light of certain limitations. These are the retrospective nature and the relatively small sample size of participants from a single center. The vast majority of patients were males, and our results cannot represent the general population. Patients that did not present clinical signs of myocardial ischemia or neurological events may not have been detected during the early follow-up. Furthermore, the short hospitalization period may hamper firm conclusions regarding the role of inflammatory markers in adverse events prediction after EVAR. The impact of AKI on the in-hospital inflammatory marker elevation cannot be excluded. Probably, a subclinical AKI during hospitalization, which may be associated with underlying renal ischemia and aseptic inflammation, may have affected the biochemical markers. However, this fact cannot be admitted or rejected from the current analysis. An analysis which would include an evaluation of the renal parenchyma could respond to the arising questions. However, such an approach is out of the scope of this study. An additional limitation is that most of the patients were discharged on day 1 after surgery. Under this spectrum, a delayed and not detected inflammatory response cannot be precluded. Additionally, we analyzed the impact of the inflammation only in early adverse events. Probably, in a more extended follow-up, the association of the inflammatory markers and adverse events would be of more interest. Nonetheless, all the data were acquired prospectively. However, it would be worth extending this study to other centers and to prospectively include patients in order to establish the prognostic value of NLR and PLR.

## 5. Conclusions

Increased postoperative NLR and PLR are associated with the occurrence of AKI after EVAR for AAA. In this subgroup of patients, closer surveillance and follow-up after EVAR may be required postoperatively in order to diagnose and treat the complications in a timely manner.

## Figures and Tables

**Table 1:** Patients’ demographic characteristics and comorbidities.

Preoperative Patients’ Characteristics	Number (%)
Age	72.1 year (range 64–82)
Males	228 (99.1)
Tobacco use	162 (70.4)
Hypertension	193 (83.9)
Dyslipidemia	183 (79.6)
CAD	101 (43.9)
COPD	118 (51.3)
DM	38 (16.5)
Renal insufficiency (GFR < 60 mL/min/1.73 m^2^)	18 (7.8)
Hemodialysis	1 (0.4)

CAD: coronary artery disease; COPD: chronic obstructive pulmonary disease; DM: diabetes mellitus; GFR: glomerular filtration rate (mL/h/1.73 m^2^).

**Table 2:** Laboratory results in patients that underwent elective EVAR.

Laboratory Findings	Mean Value (IQR Range)
Hemoglobin (mg/dL)	
Preoperative value	13.7 (10.8–16.1)
Postoperative value	11.8 (7.9–13.9)
Platelets (n/μL)	
Preoperative value	220.000 (96.000–380.000)
Postoperative value	169.000 (101.000–287.000)
NLR	
Preoperative value	3.34 (1.2–7.6)
Postoperative value	8.64 (2.4–10.2)
PLR	
Preoperative value	11.37 (3.4–20.1)
Postoperative value	17.21 (9.3–29.8)
Creatinine (mg/dL)	
Preoperative value	0.95 (0.62–3.6)
Postoperative value	0.94 (0.72–3.4)

Median preoperative NLR and PLR values were significantly increased after the procedure (NLR: from 3.34 to 8.64, *p* < 0.001 and PLR: from 11.37 to 17.21, *p* < 0.001). EVAR: endovascular aneurysm repair; NLR: neutrophil–lymphocyte ratio; PLR: platelet–lymphocyte ratio; IQR: interquartile range.

**Table 3:** All adverse events during the early follow-up in patients that underwent elective EVAR.

Postoperative Adverse Events	Number (%)
Myocardial infarction	3 (1.3)
Arrhythmia	3 (1.3)
Stroke	1 (0.4)
Limb occlusion	9 (3.9)
Infection	17 (7.4)
Surgical trauma	4 (1.7)
Respiratory system	6 (2.6)
Urinary tract	7 (3.0)
PIS	37 (16.0)
Aneurysm rupture	1 (0.4)
Renal complications	5 (2.2)
AKI (RIFLE criteria)	5 (2.2)
Hemodialysis	0 (0.0)

PIS was the most common postoperative complication followed by infections. EVAR: endovascular aneurysm repair; PIS: postimplantation syndrome; AKI: acute kidney injury; RIFLE: Risk, Injury, Failure, Loss of kidney function, and End-stage kidney disease.

## Data Availability

The data presented in this study are available on request from the corresponding author. The data are not publicly available due to ethics and general data protection regulation.

## Supplementary Materials

The following are available online at https://www.mdpi.com/2077-0383/10/5/1083/s1, Table S1: The univariate/multivariate analysis regarding the association with major cardiovascular events, Table S2: The univariate/multivariate analysis regarding the association with acute kidney injury.
