# Supplementary material for: Association of Neutrophil–Lymphocyte and Platelet–Lymphocyte Ratio with Adverse Events in Endovascular Repair for Abdominal Aortic Aneurysm
Source: J Clin Med. 2021 Mar 5;10(5):1083. doi: 10.3390/jcm10051083 (PMC7961355; doi:10.3390/jcm10051083)
Supplement: Supplementary file 1 [file jcm-10-01083-s001.pdf]

Supplementary tables

**Factors**	**HR**	**95% CI**	**p**
PLT pre-operative	0.95	0.89-1.005	0.07
PLT post-operative	1.079	0.99-1.16	0.06
PLR pre-operative	1.036	0.87-1.23	0.69
**PLR post-operative**	0.956	0.74-1.22	0.72
NLR pre-operative	0.607	0.18-2.052	0.42
**NLR post-operative**	0.956	0.909-1.216	0.42
Hgb pre-operative	3.7	0.85-1.64	0.52
Hgb post-operative	0.314	0.09-1.077	0.07
Creatinine pre-operative	0.32	0.04-32.4	0.67
Creatinine post-operative	2.8	0.02-35.21	0.63
Gender	0.98	0.016-58.4	0.99
Aneurysm diameter	1.043	0.946-1.15	0.40
Operation duration	1.015	0.993-1.037	0.19
Radiation	1.026	0.963-1.093	0.42
Median Contrast	0.998	0.969-1.216	0.90

Table S1. The univariate/multivariate analysis regarding the association with major cardiovascular events. Hgb: hemoglobin; NLR: Neutrophil - Lymphocyte Ratio; PLR: Platelet - Lymphocyte Ratio; PLT: platelets

Factors	HR	95% CI	p
PLT pre-operative	1.06	0.991-1.02	0.42
PLT post-operative	1.004	0.977-1.033	0.75
PLR pre-operative	1.079	0.956-1.218	0.21
**PLR post-operative**	**1.13**	**0.655-1.51**	**0.02**
NLR pre-operative	0.79	0.513-1.24	0.317
**NLR post-operative**	**1.178**	**0.909-1.991**	**0.01**
Hgb pre-operative	1.078	0.723-1.607	0.71
Hgb post-operative	1.045	0.679-1.609	0.841
Creatinine pre-operative	1.557	0.187-12.9	0.682
Creatinine post-operative	0.419	0.42-42.4	0.46
Gender	0.99	0.02-42.4	0.99
Aneurysm diameter	1.043	0.946-1.15	0.40
Operation duration	0.995	0.985-1.005	0.331
Radiation	0.04	0.001-0.08	0.991
Median Contrast	1.003	0.997-1.01	0.330

Table S2. The univariate/multivariate analysis regarding the association with acute kidney injury. Hgb: hemoglobin; NLR: Neutrophil - Lymphocyte Ratio; PLR: Platelet - Lymphocyte Ratio; PLT: platelets
